# Supplementary material for: Generative Multiobjective Bayesian Optimization with Scalable Batch Evaluations for Sample-Efficient De Novo Molecular Design
Source: Ind Eng Chem Res. 2025 Dec 21;65(1):628–42. doi: 10.1021/acs.iecr.5c03166 (PMC12810395; doi:10.1021/acs.iecr.5c03166)
Supplement: Supplementary file 1 [file ie5c03166_si_001.pdf]

Supplementary Information for Generative Multi-Objective Bayesian Optimization with Scalable Batch Evaluations for Sample-Efficient *De Novo* Molecular Design

Madhav R. Muthyala[a,b], Farshud Sorourifar[b], Tianhong Tan[a,b], You Peng[c], and Joel A. Paulson[a,b]

[a]University of Wisconsin–Madison, Department of Chemical and Biological Engineering, Madison, MI, 53706, USA
[b]The Ohio State University, Department of Chemical and Biomolecular Engineering, Columbus, OH, 43210, USA
[c]Chemometrics, AI and Statistics, Technical Expertise and Support, The Dow Chemical Company, Lake Jackson, Texas 77566, USA

October 22, 2025

Contents

S1 Major Classes of Generative Models **2**
S1.1 Variational autoencoders (VAEs) 2
S1.2 Autoregressive models 2
S1.3 Diffusion and flow models 2
S1.4 Evolutionary methods 3
S1.5 Reinforcement learning (RL) 3
S2 Implementation Details **3**
S2.1 Benchmark methods 3
S2.2 Our proposed method 4
S2.3 Computational time comparison 5
S3 Single-Objective Optimization of logP **5**
S3.1 Problem setup 6
S3.2 Results 6
S3.3 Discussion 6
S4 Comparison to REINVENT **7**
S4.1 Multi-objective optimization of penalized logP and TPSA 7
S4.2 Design of organic electrode materials 8

S1 Major Classes of Generative Models

Stage 1 in our framework is intentionally *generator-agnostic*: any method capable of producing a large pool of valid molecules can plug in, and Stage 2 handles the multi-objective down-selection. Below we review the main classes of molecular generators, how they function, and the trade-offs they bring in terms of sample efficiency, validity, controllability, and coupling to surrogate models. Our aim is not to prescribe a winner but to clarify when each approach is most appropriate and what limitations often motivate our decoupled "generate-then-optimize" design.

S1.1 Variational autoencoders (VAEs)

VAEs embed discrete molecule representations into a continuous latent space, then decode latent vectors back into molecular structures [1]. In training, the encoder and decoder are jointly optimized via a reconstruction loss plus a Kullback-Leibler (KL) divergence regularizer; property conditioning can be added via auxiliary predictors or conditional VAE variants [2]. Once trained, new molecules are generated by sampling latent vectors and decoding them.

VAEs offer smooth latent interpolations and support gradient-based navigation in latent space, which is appealing when optimizing a single (or scalarized) objective. But in practice, the continuous latent space may not align well with discrete chemical structure, making surrogate modeling and uncertainty quantification difficult, especially in multi-objective settings. Decoding may yield invalid or redundant molecules, and batch acquisition in latent space often must rely on heuristics. Some works tackle this via local BO in latent space or trust-region strategies [3]. In our pipeline, VAEs are best deployed as fast proposers of diverse candidates; Stage 2 then handles the rigorous multi-objective ranking without requiring joint training of encoder, decoder, and surrogate. Empirically, VAE-based generators often underperform compared to alternatives in multi-objective molecule discovery [4], but they remain useful baselines and fallback options.

S1.2 Autoregressive models

Autoregressive models generate molecules one token or one graph action at a time. For example, when using SMILES/SELFIES strings, each token is predicted conditioned on previous tokens. Alternatively, graph-based autoregressive models decide on atoms, bonds, or edges step-by-step. Training typically uses maximum likelihood over known molecules; conditioning is introduced via fine-tuning or conditioning prompts.

These models are strong at validity and language-like sequence modeling. They can easily incorporate domain priors via prompts or context, but they are sensitive to representation choices (e.g. SMILES canonicalization), suffer from exposure bias (errors propagate during generation), and often stay close to training distributions without stronger exploration forces. In tightly coupled schemes, autoregressive models may over-optimize toward surrogate objectives, reducing diversity.

In our framework, autoregressive models act as scalable, high-validity proposers: they can rapidly emit large pools of structurally plausible candidates biased toward relevant chemical spaces. Stage 2 then intervenes to restore principled trade-offs by selecting the subset that maximizes expected Pareto improvement. This decoupling helps avoid overfitting generation to the surrogate.

S1.3 Diffusion and flow models

Diffusion models generate molecules by progressively denoising a random latent through a learned transition, whereas normalizing flows map molecules and latent vectors via invertible transformations. Both approaches support conditioning (e.g. score-based guidance in diffusion, conditional flows) and tend to produce diverse, high-quality samples, including geometry-aware variants for three-dimensional molecular structures [5]. Flow-autoregressive hybrids (e.g. GraphAF [6]) combine the benefits of flows and autoregressive sampling with validity checks during generation.

These models excel when sample quality and diversity matter most, and when geometric consistency or symmetry is important. However, training and sampling can be more compute-intensive, and coupling them tightly to property surrogates risks over-optimization. In our generate-first paradigm, diffusion/flow models

can generate a broad structural scaffold in bulk (often in unconditional or lightly guided mode), leaving Stage 2 to impose multi-objective constraints and select the most promising candidates.

S1.4 Evolutionary methods

Evolutionary algorithms (EAs) evolve populations of molecules via mutation, crossover, and selection directly in chemical space. A prominent example is Graph-GA [7], which has been demonstrated to traverse structural space effectively (e.g., discovering novel scaffolds in logP optimization tasks). More recent work highlights how GAs remain strong baselines [8]. In multi-objective molecular optimization, variants of NSGA-II and NSGA-III have been adapted to work over molecular graphs, enforcing diversity and Pareto advancement without relying on learned models [9]

Graph-GA is attractive because it is simple, interpretable, and fairly flexible in terms of the graph representation. It naturally incorporates domain heuristics (e.g. synthesizability filters, substructure constraints) and does not require training. However, it can be evaluation-inefficient and prone to local minima if diversity is not carefully enforced. In our framework, Graph-GA can serve as an adaptive proposer: we could bias mutation/selection pressures toward molecules in regions of high surrogate uncertainty or predicted potential, then hand off the candidate set to Stage 2, which guards against premature convergence by selecting across the population in a multi-objective, uncertainty-aware fashion.

S1.5 Reinforcement learning (RL)

RL treats molecular generation or editing as a sequential decision problem: at each step the agent chooses an action (e.g. add atom, bond, transform fragment), receives a reward based on predicted properties (or multi-objective scalarization), and updates its policy. Early molecular RL methods include REINVENT [10], which applies policy gradient to sequence-based generators (SMILES), and MolDQN [11], which frames editing actions in a Q-learning framework. More advanced methods also combine autoregressive or flow models with RL or GFlowNet-style training for better diversity and sampling properties. GFlowNets (generative flow networks) aim to sample terminal molecules in proportion to a reward distribution, thus inherently promoting diversity instead of greedily chasing the highest reward [12]. Recent variants have incorporated goal-conditioning and multi-objective extensions [13].

RL and GFlowNets offer fine-grained control, curriculum-style learning, and constraint enforcement; however, they tend to require many reward evaluations, careful reward shaping, and suffer from instability or mode collapse. In our decoupled setup, RL or GFlowNet agents can be used to enrich regions of interest (e.g., via short rollouts targeted to promising areas) while Stage 2 maintains rigorous uncertainty-driven batch selection, limiting overfitting of the surrogate.

S2 Implementation Details

S2.1 Benchmark methods

For the initial case studies, we compare our proposed method (described next) to four alternative methods. Each of these baselines represents a different family of generative optimization techniques. Below, we describe their implementation and how we adapt them for the multi-objective setting:

- **VAE+BO** [1]: This approach follows the latent-optimize-then-decode framework introduced by Gómez-Bombarelli et al., where molecules are embedded into a continuous latent space using a VAE, optimized with BO, and then decoded back into molecular structures. We implement the same architecture as the original paper, with a sparse GP surrogate model and the qLogEI acquisition function (for single-objective problems) or qLogEHVI (for multi-objective problems), both implemented using `BoTorch` [14]. LogEI and LogEHVI offer numerically stable approximations of Expected Improvement (EI) and Expected Hypervolume Improvement (EHVI), respectively, that are well-suited for multi-point (batch) optimization [15].

- **JANUS** [16]: JANUS is a parallel-tempered GA that integrates a neural network property predictor to guide evolutionary selection. It uses multiple interacting populations at different "temperatures"

to balance exploration and exploitation. We adopt the default configuration and hyperparameters provided in the original implementation, available at https://github.com/aspuru-guzik-group/JANUS. As JANUS is designed for single-objective optimization, we extend it to multi-objective settings by optimizing a composite scalar objective defined as a weighted sum of the normalized objectives, with weights determined based on min-max scaling of the initial dataset.

- **Graph GA** [7]: This method is a graph-based GA that builds molecules through mutations and crossovers of molecular fragments. It starts with a seed population of valid molecules and applies chemically informed transformations, using a molecular graph representation and `RDKit` sanitization to enforce valency and stability constraints. The fitness function is based on a target property and selection favors molecules with improved performance. We use the default configuration and hyperparameters described in the original paper and implementation, available at https://github.com/jensengroup/GB_GA. To adapt Graph GA to multi-objective problems, we again apply a scalarization strategy using min-max normalized objectives and fixed weights.

- **MolDQN** [11]: MolDQN is a deep reinforcement learning method based on Q-learning that incrementally edits molecular graphs to optimize desired properties. It models the problem as a Markov decision process, where each action corresponds to a chemical transformation. The policy is trained to maximize a reward function, which we take as a scalarized version of the multi-objective targets. We use the original implementation and training procedure from https://github.com/google-research/google-research/tree/master/mol_dqn, with no changes to architecture or hyperparameters. For multi-objective problems, we define the reward as a linear combination of normalized objectives using fixed weights (same as JANUS and Graph GA).

Constraint handling: For case studies where structural constraints are required to prevent trivial solutions (e.g., overly long SMILES strings in logP optimization), we incorporate filtering or rejection mechanisms specific to each method. In VAE+BO, we inject increasing Gaussian noise into the latent vector until a decoded molecule satisfies the constraints. For JANUS and Graph GA, we apply post-generation filtering on the candidate pool before the final batch selection to be sent to oracle evaluation. For MolDQN, we regenerate batches until a valid set of constraint-satisfying molecules is found.

The full implementation details, including hyperparameters and evaluation pipelines for all baseline methods, are available in our code repository: https://github.com/PaulsonLab/Generative_MOBO_qPMHI.

S2.2 Our proposed method

Our approach follows a modular two-stage design framework. Stage 1 involves the generative proposal of candidate molecules, while Stage 2 performs surrogate-based batch selection using our novel acquisition function (i.e., qPMHI).

Stage 1: Generative proposal. We explore several generator types to highlight both the flexibility of our framework and the role of the generator in optimization performance. Our default choice is a lightweight genetic algorithm (GA) applied to the SELFIES representation of molecules, guided by the surrogate model used in Stage 2. Let $\widehat{\boldsymbol{f}} : \mathcal{X} \to \mathbb{R}^M$ and $\boldsymbol{\Sigma} : \mathcal{X} \to \mathbb{R}^{M \times M}$ denote the surrogate's predictive mean and covariance functions. Candidates are generated by maximizing a composite scoring function:

$$J_{\boldsymbol{\lambda}}(\boldsymbol{s}; \widehat{\boldsymbol{f}}, \boldsymbol{\Sigma}, \boldsymbol{g}),$$

where $\boldsymbol{s}$ is a SELFIES string, $\boldsymbol{g}$ denotes optional auxiliary metrics (e.g., synthesizability), and $\boldsymbol{\lambda}$ is a tunable parameter vector that balances the components of the score. Rather than fixing $\boldsymbol{\lambda}$, we sweep over many random instantiations (typically drawing each coefficient uniformly between 3 and 5) and aggregate the resulting unique molecules across GA runs. This ensemble-style approach encourages candidate diversity and balances exploitation (high predicted utility) with exploration (uncertain regions or auxiliary objectives). We implement the GA using the `DEAP` Python package [17], following the STONED methodology [18], which leverages the robustness of SELFIES to avoid invalid molecule generation.

In addition to the default GA, we evaluate two alternative generators. The first is a VAE-based generator, reusing the same trained model from the VAE+BO baseline. Here, we sample latent points in the

Table S.1: Wall-clock time per optimization iteration (mean ± std, seconds) on the logP-TPSA study. Note that these methods were not all run on the same computing cluster or optimized for speed, so results should be viewed as approximate indicators.

Method	Time per iteration (min)
Ours†	11.71 ± 0.10
JANUS†	12.63 ± 0.57
VAE+BO‡	11.95 ± 0.52
VAE+GP‡	11.75 ± 0.05
MolDQN‡	11.44 ± 0.05
Graph-GA§	2.21 ± 0.03

†Run on computer with 16 CPU cores, 512 GB RAM.
‡Run on computer with 16 CPU cores, 512 GB RAM, NVIDIA A100 (40 GB).
§Run on computer with 48 CPU cores, 192 GB RAM; heavily parallelized run.

neighborhood of those proposed by BO and decode them into molecules. The second is a REINVENT-style generator, which uses a recurrent neural network trained via reinforcement learning to propose molecules with favorable properties (see Section S4 for details). For all generators, if the initial candidate pool does not meet any potential desired size or constraint requirements, we re-run the generator with new random seeds until a sufficiently large, valid pool is obtained.

Stage 2: Surrogate modeling and batch selection. For Stage 2, we develop a custom Bayesian graph neural network (BGNN) surrogate model. Each molecule is represented as a molecular graph, parsed from its SMILES string, with atom-level node features and bond-level edge features. The encoder is a deterministic graph attention network with message passing, adapted from Ramani and Karmakar [19], which maps each graph to a 200-dimensional latent feature vector. This embedding is passed through a series of Bayesian linear layers with ReLU activations to produce property predictions.

The BGNN is trained via variational inference to minimize the Kullback-Leibler (KL) divergence between the true posterior and a tractable variational approximation. Optimization is performed using the AdamW optimizer with a learning rate of 0.001. We use the `torchbnn` package to implement the variational layers, and apply the post-hoc uncertainty calibration method proposed by Rasmussen et al. [20] to improve predictive variance estimation. For computational efficiency, we update the BGNN parameters every 5 optimization iterations, with each update using 100 training epochs (i.e., full passes through the dataset).

S2.3 Computational time comparison

Table S.1 reports the (end-to-end) wall-clock time per optimization iteration for the multi-objective logP-TPSA case study (reported in Section 4 of the main body). Values are mean ± standard deviation (minutes), computed across iterations of all replicates. Two caveats are important for interpreting these numbers. First, due to practical constraints on software installation and cluster availability, not all baselines were executed on identical hardware; we indicate the configuration for each method in the table footnote. Second, we did not tune any code for speed. For the applications we target – where the oracle is a high-fidelity simulation or an experiment – minutes per iteration are typically negligible compared to evaluation time, which can be hours to days or longer. Nevertheless, scenarios with cheap oracles may warrant additional engineering; we view systematic runtime optimization as future work.

S3 Single-Objective Optimization of logP

We assess the generate-then-optimize framework on a single-objective benchmark to enable direct comparison with widely used baselines that are primarily tailored for single-objective settings.

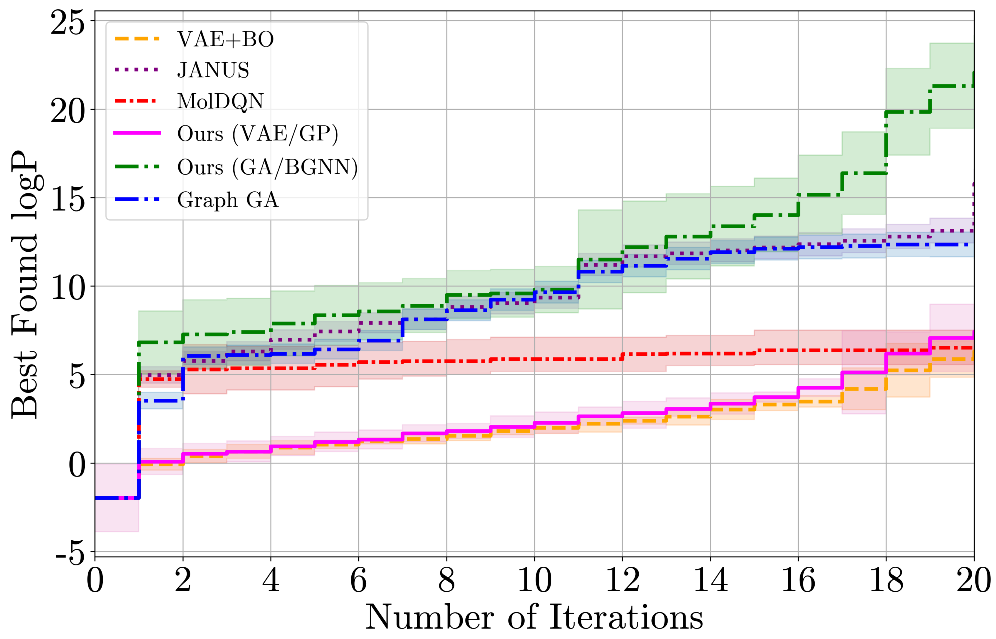

Figure S.1: Single-objective logP optimization results across 20 iterations (batch size $q = 50$). Curves show mean best-found logP across five seeds; bands denote 95% confidence intervals. Our method, *Ours (GA/BGNN)* (green), consistently outperforms all baselines.

S3.1 Problem setup

We maximize the water–octanol partition coefficient (logP), following the Gómez-Bombarelli et al. setup [1]. Molecules are drawn from ZINC-250k [21] and logP is computed using the Crippen method [22] via `RDKit` [23]. To avoid trivial "hacks" that are known to exist for logP, we enforce two constraints throughout: SMILES length ≤ 108 and SAScore ≤ 8 (which is a measure of the ease of synthesizing a molecule).

All methods are implemented as in Section S2. We run $T = 20$ iterations with batch size $q = 50$. For our method, we use $L = 256$ Monte Carlo samples to compute acquisition values and set the Stage 1 pool size to $N = 5000$. The initial training set $\mathcal{D}_0$ contains 2000 randomly sampled, labeled molecules from ZINC-250k. Each method is run with five random seeds; the same five seeds are used across methods to ensure consistency.

S3.2 Results

Figure S.1 shows the best-found logP versus iteration for six methods (means over five seeds with 95% confidence intervals). Our approach, *Ours (GA/BGNN)*, using qPO for single-objective selection, improves most rapidly and attains the highest final values (exceeding 20 on average). The next strongest methods are the two GA families: *JANUS* and the newly added *Graph-GA*, which track closely and plateau near the mid-teens by iteration 20. *MolDQN* and the two VAE-based baselines remain substantially lower. Table S.2 reports the final best-found logP for each seed. Our method is best on every replicate. As a point of reference for the Stage 2 effect, *Ours (VAE/GP)*, which retains the same VAE generator but uses our two-stage selection, improves noticeably over *VAE+BO*.

S3.3 Discussion

Two observations stand out. First, *JANUS* and *Graph-GA* (both GA-based) perform in the same band and above MolDQN and the VAE-based baselines. *Graph-GA* averages 14.93 ± 0.32 across seeds, while *JANUS* averages 15.78 ± 0.29, consistent with the side-by-side trajectories in Figure S.1. This suggests that evolutionary search with chemically informed operators and diversity pressure is an effective strategy on

Table S.2: Final best-found logP across five independent runs (one per seed). The Graph-GA baseline was newly added and performs similarly to JANUS. The best value in each row is underlined.

Run	VAE+BO	JANUS	Graph-GA	MolDQN	Ours (VAE/GP)	Ours (GA/BGNN)
1	6.21	16.14	14.66	6.68	7.21	<u>25.26</u>
2	6.15	15.78	15.10	6.06	7.64	<u>24.09</u>
3	6.49	15.41	15.30	6.53	7.59	<u>16.32</u>
4	6.30	15.96	15.05	6.96	7.24	<u>19.92</u>
5	6.44	15.60	14.54	6.37	7.50	<u>24.82</u>

this benchmark. Second, our two-stage method delivers a larger and more consistent improvement: *Ours (GA/BGNN)* averages 22.08 ± 3.86, with fast early gains and several large jumps later in the run. The *Ours (VAE/GP)* versus *VAE+BO* comparison isolates the contribution of the selection stage: holding the VAE generator fixed while switching to our acquisition-driven, batchwise selection yields a clear boost (from 6.32 ± 0.15 to 7.44 ± 0.20 on average), underscoring the value of decoupling generation from objective-driven selection even with imperfect generators.

Overall, the single-objective results reinforce two principles that motivate the full framework: (i) the generator matters, with GA families being strong in this application and (ii) principled, acquisition-based selection confers additional gains beyond generator choice, which becomes critical as we transition to *de novo* multi-objective problems.

S4 Comparison to REINVENT

To further understand the generalizability of our proposed two-stage generate-then-optimize framework, we conducted additional experiments comparing it against REINVENT [10], a widely used RL-based molecular generator. REINVENT has demonstrated strong performance (ranked number 1 out of the tested options) in prior benchmarks for molecular property optimization [4]. These comparisons allow us to better assess the complementarity and robustness of our proposed qPMHI-based selection strategies when paired with different generative models, as well as to highlight challenges that arise in distributional mismatch between training and target domains.

S4.1 Multi-objective optimization of penalized logP and TPSA

REINVENT uses a recurrent neural network (RNN) over SMILES strings, trained initially on ~1.5M canonical molecules from the ChEMBL database [24]. During optimization, it performs policy-gradient updates using an augmented likelihood:

$$\log P_{\text{aug}}(\boldsymbol{x}) = \log P_{\text{prior}}(\boldsymbol{x}) + \sigma S(\boldsymbol{x}),$$

where $S(\boldsymbol{x})$ denotes a task-specific score and the tuning parameter σ balances exploration and exploitation. To ensure a fair comparison, we reuse the same REINVENT-trained prior and focus on a two-objective task: maximize penalized logP (standard logP minus a synthetic accessibility score and ring penalty) and minimize topological polar surface area (TPSA). Note that this is slightly different than the constrained logP studied in the main body and Section S3.

Setup. We warm-start both methods with 10 iterations of REINVENT using its default parameters ($\sigma = 128$, learning rate 5×10^{-4}) and a batch size $q = 128$. After this shared phase, we train a surrogate GP model using Extended Connectivity FingerPrints (ECFP) (radius 3) with a Tanimoto kernel [25, 26]. For iterations 11 onward, REINVENT continues standard RL fine-tuning, while our method applies qPMHI selection to a large oversampled pool of 5000 SMILES generated from REINVENT rollouts (100 rollouts of length 50 on the surrogate mean). This ensures both methods operate in the same molecular prior space.

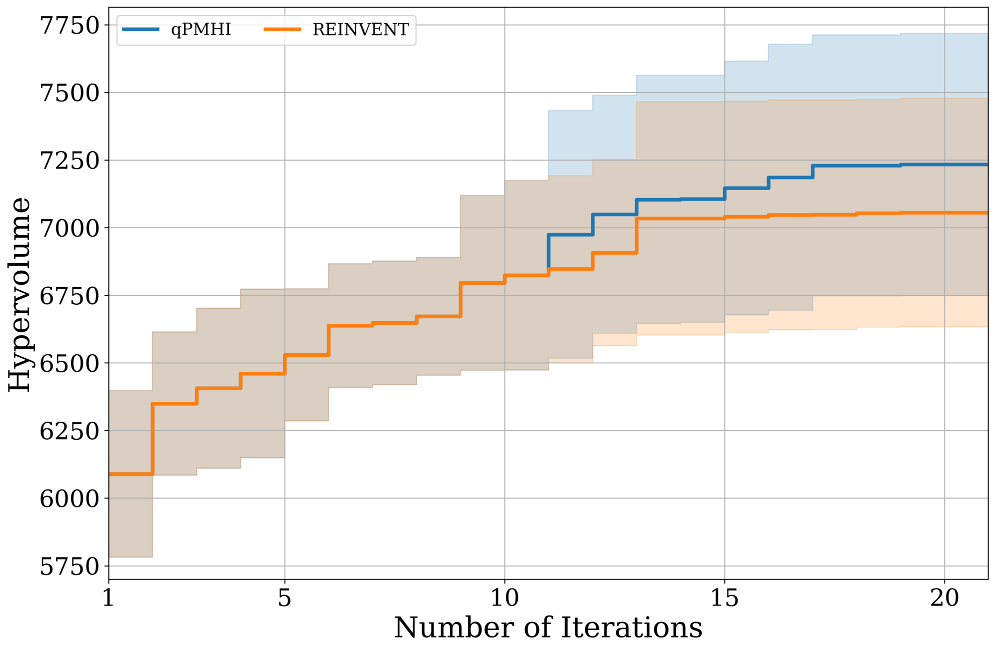

Figure S.2: Pareto front hypervolume over 20 optimization iterations on the penalized logP-TPSA benchmark. Both methods share the same REINVENT-generated prior and are identical for the first 10 iterations. From iteration 11 onward, the proposed method (qPMHI) applies surrogate-based batch selection on a large REINVENT-generated pool. Curves show the mean over 5 runs; shaded regions indicate 95% confidence intervals. Results show that augmenting REINVENT with qPMHI consistently improves performance.

Results. Figure S.2 presents the hypervolume of the Pareto front versus number of iterations, averaged across five independent runs. After the initial shared phase, switching to qPMHI produces a sharp increase in performance at iteration 11, and this advantage is maintained through iteration 20. Across iterations 12-20, our method consistently outperforms REINVENT by 100–200 hypervolume units, with the qPMHI confidence bands remaining elevated throughout. These results show that REINVENT, while capable of generating diverse candidates, can clearly benefit from an uncertainty-aware selection mechanism. This further reinforces the central idea behind our framework: that generator-agnostic optimization via acquisition-guided selection offers a principled and effective way to enhance performance.

S4.2 Design of organic electrode materials

We also tested REINVENT on our more challenging multi-objective design task involving organic electrode materials (OEMs), where the goal is to jointly optimize redox potential and aqueous solubility. Unlike the other benchmark problems focused on drug-like molecules, this task involves more electrochemically diverse structures from a quinone-rich design space curated by Tabor et al. [27]. As before, we use the default REINVENT configuration and the same ChEMBL-trained prior model. However, this introduces a distributional mismatch: the ChEMBL prior is heavily biased toward small bioactive molecules, while the OEM library contains larger, more functionalized redox-active species.

Results. Figure S.3 plots hypervolume progression for REINVENT over 100 iterations with batch size 128. While REINVENT explores a number of molecules, its final Pareto front reaches a Pareto front hypervolume of only $\sim$1.34, which is substantially worse than the initial $\mathcal{D}_0$ (14.89) used in our main experiments. These results confirm that REINVENT, in its default form, fails to make meaningful progress in this application. While we expect that retraining the prior on a more relevant dataset would substantially improve performance, that is beyond the scope of this study. Nonetheless, this experiment highlights an important takeaway: the alignment between generator training distribution and target design space plays a crucial role

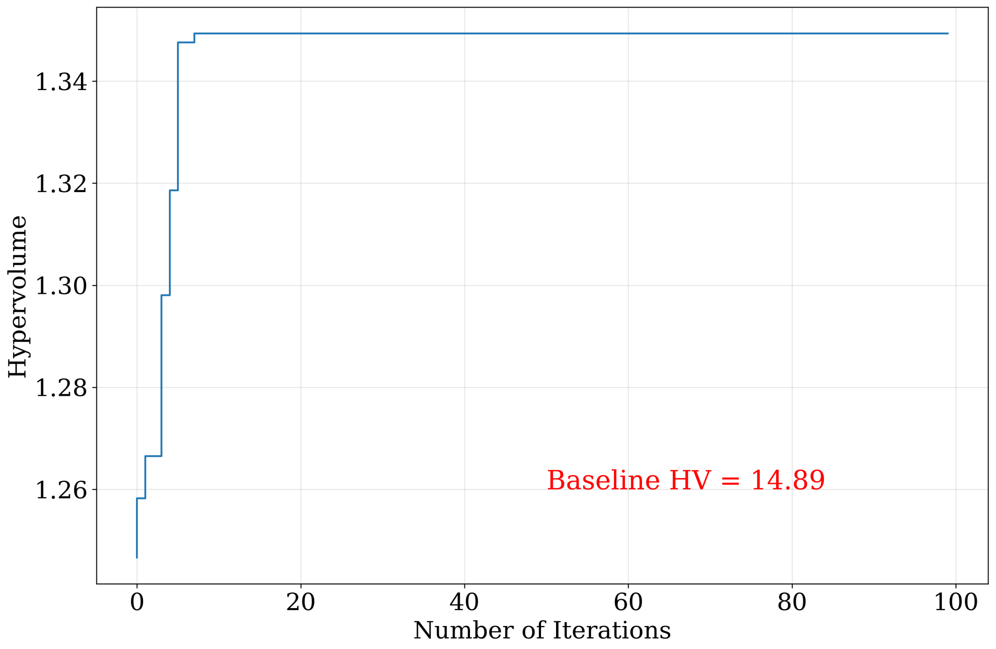

Figure S.3: Performance of REINVENT on the multi-objective organic electrode material (OEM) design task. Shown is the Pareto front hypervolume across 100 iterations (batch size 128) for a single run. The red label indicates the baseline hypervolume (14.89) of the initial set $\mathcal{D}_0$ used in the main paper. Despite running for many iterations, REINVENT fails to improve over the initial dataset due to poor alignment between its ChEMBL-trained prior and the quinone-rich OEM design space.

in downstream optimization outcomes and should be carefully considered when selecting generative models for molecular discovery.

References

[1] Rafael Gómez-Bombarelli, Jennifer N Wei, David Duvenaud, José Miguel Hernández-Lobato, Benjamín Sánchez-Lengeling, Dennis Sheberla, Jorge Aguilera-Iparraguirre, Timothy D Hirzel, Ryan P Adams, and Alán Aspuru-Guzik. Automatic chemical design using a data-driven continuous representation of molecules. *ACS Central Science*, 4(2):268–276, 2018.

[2] Jaechang Lim, Seongok Ryu, Jin Woo Kim, and Woo Youn Kim. Molecular generative model based on conditional variational autoencoder for de novo molecular design. *Journal of Cheminformatics*, 10(1):31, 2018.

[3] Natalie Maus, Haydn Jones, Juston Moore, Matt J Kusner, John Bradshaw, and Jacob Gardner. Local latent space bayesian optimization over structured inputs. In S. Koyejo, S. Mohamed, A. Agarwal, D. Belgrave, K. Cho, and A. Oh, editors, *Advances in Neural Information Processing Systems*, volume 35, pages 34505–34518. Curran Associates, Inc., 2022.

[4] Wenhao Gao, Tianfan Fu, Jimeng Sun, and Connor Coley. Sample efficiency matters: A benchmark for practical molecular optimization. *Advances in Neural Information Processing Systems*, 35:21342–21357, 2022.

[5] Alex Morehead and Jianlin Cheng. Geometry-complete diffusion for 3D molecule generation and optimization. *Communications Chemistry*, 7(1):150, 2024.

[6] Chence Shi, Minkai Xu, Zhaocheng Zhu, Weinan Zhang, Ming Zhang, and Jian Tang. Graphaf: A flow-based autoregressive model for molecular graph generation. *arXiv preprint arXiv:2001.09382*, 2020.

[7] Jan H Jensen. A graph-based genetic algorithm and generative model/Monte Carlo tree search for the exploration of chemical space. *Chemical Science*, 10(12):3567–3572, 2019.

[8] Austin Tripp and José Miguel Hernández-Lobato. Genetic algorithms are strong baselines for molecule generation. *arXiv preprint arXiv:2310.09267*, 2023.

[9] Jonas Verhellen. Graph-based molecular Pareto optimisation. *Chemical Science*, 13(25):7526–7535, 2022.

[10] Marcus Olivecrona, Thomas Blaschke, Ola Engkvist, and Hongming Chen. Molecular de-novo design through deep reinforcement learning. *Journal of Cheminformatics*, 9:1–14, 2017.

[11] Zhenpeng Zhou, Steven Kearnes, Li Li, Richard N Zare, and Patrick Riley. Optimization of molecules via deep reinforcement learning. *Scientific Reports*, 9(1):10752, 2019.

[12] Yoshua Bengio, Salem Lahlou, Tristan Deleu, Edward J Hu, Mo Tiwari, and Emmanuel Bengio. Gflownet foundations. *Journal of Machine Learning Research*, 24(210):1–55, 2023.

[13] Yiheng Zhu, Jialu Wu, Chaowen Hu, Jiahuan Yan, kim hsieh, Tingjun Hou, and Jian Wu. Sample-efficient multi-objective molecular optimization with gflownets. In A. Oh, T. Naumann, A. Globerson, K. Saenko, M. Hardt, and S. Levine, editors, *Advances in Neural Information Processing Systems*, volume 36, pages 79667–79684. Curran Associates, Inc., 2023.

[14] Maximilian Balandat, Brian Karrer, Daniel Jiang, Samuel Daulton, Ben Letham, Andrew G Wilson, and Eytan Bakshy. BoTorch: A framework for efficient Monte-Carlo Bayesian optimization. *Advances in Neural Information Processing Systems*, 33:21524–21538, 2020.

[15] Sebastian Ament, Samuel Daulton, David Eriksson, Maximilian Balandat, and Eytan Bakshy. Unexpected improvements to expected improvement for bayesian optimization. *Advances in Neural Information Processing Systems*, 36:20577–20612, 2023.

[16] AkshatKumar Nigam, Robert Pollice, and Alán Aspuru-Guzik. Parallel tempered genetic algorithm guided by deep neural networks for inverse molecular design. *Digital Discovery*, 1(4):390–404, 2022.

[17] Félix-Antoine Fortin, François-Michel De Rainville, Marc-André Gardner, Marc Parizeau, and Christian Gagné. DEAP: Evolutionary algorithms made easy. *Journal of Machine Learning Research*, 13:2171–2175, jul 2012.

[18] AkshatKumar Nigam, Robert Pollice, Mario Krenn, Gabriel dos Passos Gomes, and Alan Aspuru-Guzik. Beyond generative models: superfast traversal, optimization, novelty, exploration and discovery (STONED) algorithm for molecules using SELFIES. *Chemical Science*, 12(20):7079–7090, 2021.

[19] Vansh Ramani and Tarak Karmakar. Graph neural networks for predicting solubility in diverse solvents using molmerger incorporating solute–solvent interactions. *Journal of Chemical Theory and Computation*, 20(15):6549–6558, 2024.

[20] Maria H Rasmussen, Chenru Duan, Heather J Kulik, and Jan H Jensen. Uncertain of uncertainties? a comparison of uncertainty quantification metrics for chemical data sets. *Journal of Cheminformatics*, 15(1):121, 2023.

[21] John J Irwin, Teague Sterling, Michael M Mysinger, Erin S Bolstad, and Ryan G Coleman. ZINC: A free tool to discover chemistry for biology. *Journal of Chemical Information and Modeling*, 52(7):1757–1768, 2012.

[22] Scott A Wildman and Gordon M Crippen. Prediction of physicochemical parameters by atomic contributions. *Journal of Chemical Information and Computer Sciences*, 39(5):868–873, 1999.

[23] RDKit: Open-source cheminformatics software. `https://www.rdkit.org`. Accessed: July 17, 2025.

[24] Anna Gaulton, Louisa J. Bellis, A. Patricia Bento, Jon Chambers, Mark Davies, Anne Hersey, Yvonne Light, Shaun McGlinchey, David Michalovich, Bissan Al-Lazikani, and John P. Overington. Chembl: a large-scale bioactivity database for drug discovery. *Nucleic Acids Research*, 40(D1):D1100–D1107, 09 2011.

[25] David Rogers and Mathew Hahn. Extended-connectivity fingerprints. *Journal of Chemical Information and Modeling*, 50(5):742–754, 2010.

[26] Ryan-Rhys Griffiths, Leo Klarner, Henry Moss, Aditya Ravuri, Sang Truong, Yuanqi Du, Samuel Stanton, Gary Tom, Bojana Rankovic, Arian Jamasb, Aryan Deshwal, Julius Schwartz, Austin Tripp, Gregory Kell, Simon Frieder, Anthony Bourached, Alex Chan, Jacob Moss, Chengzhi Guo, Johannes Peter Dürholt, Saudamini Chaurasia, Ji Won Park, Felix Strieth-Kalthoff, Alpha Lee, Bingqing Cheng, Alan Aspuru-Guzik, Philippe Schwaller, and Jian Tang. Gauche: A library for gaussian processes in chemistry. In A. Oh, T. Naumann, A. Globerson, K. Saenko, M. Hardt, and S. Levine, editors, *Advances in Neural Information Processing Systems*, volume 36, pages 76923–76946. Curran Associates, Inc., 2023.

[27] Daniel P Tabor, Rafael Gómez-Bombarelli, Liuchuan Tong, Roy G Gordon, Michael J Aziz, and Alán Aspuru-Guzik. Mapping the frontiers of quinone stability in aqueous media: Implications for organic aqueous redox flow batteries. *Journal of Materials Chemistry A*, 7(20):12833–12841, 2019.
